# Bonwill’s Method of Anæsthesia

**Published:** 1876-08

**Authors:** P. D. Keyser


### Bonwill's Method of Anæsthesia in Diseases of the eye.

By P. D. Keyser, M. D.

It is a well known fact that in ophthalmology there are many little operations of a painful nature that occur almost daily in hospital as well as in private practice, which are really too quickly performed to require the use of an anæsthetic, but which in many cases, from the great pain and sensitiveness of the patient, cannot be made without it; and in many, if not the majority of cases, there is such an after-sickness and depression from the use of ether or chloroform that it seems very hard that one should suffer so long with this dreadful nausea for a little affair that could be accomplished in a few seconds without any after-suffering, if the patient could only hold still for a moment.

How grateful, therefore, should we feel for the suggestion of anything to take the place of ether or chloroferm in dulling sensation in cases of small operations, where complete anæsthesia is hardly necessary.

Some considerable time ago, my friend Dr. W. G. A. Bonwill, of this city, related to me his experience with the use of air as an anæsthesic in dental operations, and asked me to try it in my practice; since which time I have tried it with very happy results on many occasions. My experience with it has been, however, entirely in small or rather short but painful operations, as its anæsthetic action is not long enough for larger or more tedious manipulations.

Foreign bodies in the eye are of very frequent occurrence, and in children and nervous people, who will not remain quiet, very difficult of removal. In such cases I have found that by getting the patient to make deep and quick inspirations of the ordinary air of the room for a few seconds to a minute or so, the eye can be opened without difficulty, and the foreign body removed from the cornea or under the lid, with ease, and without the least pain or inconvenience to the patient.

In cases of painful hordeolum, which require to be opened, it affords a most happy assistance.

In slitting up a canaliculus, as well as in the introduction of Bowman's probes, it is equally efficient.

It is to my mind one of the simplest and at the same time one of the most beneficial agents in small operations about the eye that has been presented to the profession; its application being very easy, requiring no recumbent position on the part of the patient, calling for no apparatus for its administration, and being perfectly free from any of the disagreeable effects of ether and chloroform.

To produce the proper effect the patient must open his mouth, breath freely, quickly and deeply, and after a few seconds or minutes of such steady and continuous breathing, the symptoms of partial anæsthesia supervene, as is evinced by the absence of feeling in pinching or pricking with a pin. At this stage any operation should be made. The anæsthetic feeling passes almost immediately away, and the patient feels no pain in the operation if done dexterously and without hesitation.

From my experience I take pleasure in recommending its use very highly, not only to ophthalmologists, but also to the general surgeon, where minor operations are to be performed.

Any one interested in the subject I would refer to an article by Bonwill on "Air as a an Anæsthetic," in the *Pennsylvania Journal of Dental Science.*"—*Philadelphia Medical Times.*

